# Supplementary material for: Resolving the full spectrum of human genome variation using Linked-Reads
Source: Genome Res. 2019 Apr;29(4):635–45. doi: 10.1101/gr.234443.118 (PMC6442396; doi:10.1101/gr.234443.118)
Supplement: Supplemental Material [file supp_gr.234443.118_Supplemental_longranger-2.2.zip › longranger-2.2.tar.bz2!/lr2.2-public/lib/go/src/code.google.com/vitess/examples/demo/result.html]

##### {{curResult.title}}

last\_id: {{res.lastrowid}}

{{field[0]}}

{{cell}}

rows\_affected: {{curResult.rowcount}} last\_id: {{curResult.lastrowid}}

{{curResult.title}}: {{curResult.error}}
